# Elevated inflammatory markers are associated with impaired bone health in patients with adult-onset Still’s disease: A cross-sectional study

**DOI:** 10.1097/MD.0000000000050053

**Published:** 2026-07-31

**Authors:** Yong Jun Choi, Ji-Won Kim, Ju-Yang Jung, Chang-Hee Suh, Hyoun-Ah Kim

**Affiliations:** a Department of Endocrinology and Metabolism, Ajou University School of Medicine, Suwon, South Korea; b Department of Rheumatology, Ajou University School of Medicine, Suwon, South Korea.

**Keywords:** adult-onset Still’s disease, bone mineral density, C-reactive protein, ferritin, inflammation, osteoporosis, trabecular bone score

## Abstract

Adult-onset Still’s disease (AOSD) is a chronic systemic inflammatory disorder with sustained elevation of pro-inflammatory cytokines and frequent glucocorticoid use. Real-world data on bone health in AOSD remain scarce. This study aimed to evaluate bone health parameters and their associations with inflammatory markers in patients with AOSD. This cross-sectional study included 54 patients with AOSD who underwent dual-energy x-ray absorptiometry (DXA) at a tertiary referral center. Bone mineral density (BMD) at the lumbar spine, femoral neck, and total hip, and trabecular bone score (TBS) were assessed. Associations between C-reactive protein (CRP), ferritin, cumulative glucocorticoid dose, and bone parameters were analyzed using Spearman correlation, partial correlation adjusted for age, sex, and body mass index (BMI), and multivariable logistic regression. The mean age was 58.2 ± 12.0 years (79.6% female). Osteoporosis and osteopenia were present in 40.7% and 38.9%, respectively. TBS assessment (n = 52) revealed that 38 patients (73.1%) had normal microarchitecture (TBS > 1.350) and 14 (26.9%) had partially degraded microarchitecture (TBS 1.200–1.350). CRP showed a significant negative correlation with TBS (*r* = −0.445, *P* = .001), which remained significant after Bonferroni correction (adjusted *P* = .019) and after partial correlation adjusting for age, sex, and BMI (*r* = −0.336, *P* = .015). In multivariable logistic regression adjusted for age, sex, and BMI, CRP was independently associated with osteoporosis (odds ratio [OR] 1.13, 95% confidence interval [CI] 1.01–1.27, *P* = .039). In patients aged ≥ 50 years, log-transformed ferritin was independently associated with osteoporosis (OR 4.34, 95% CI 1.43–13.13, *P* = .009). Cumulative glucocorticoid dose was not significantly associated with bone parameters. These real-world data demonstrate a high burden of impaired bone health in patients with AOSD. CRP was independently associated with TBS and osteoporosis after adjustment for age, sex, and BMI, and ferritin was associated with osteoporosis in patients aged ≥50 years. These findings support the need for routine bone health screening in AOSD.

## 1. Introduction

Adult-onset Still’s disease (AOSD) is a rare systemic inflammatory disorder characterized by quotidian fever, an evanescent rash, arthritis, and markedly elevated inflammatory markers.^[1,2]^ Because its clinical features overlap with those of infections, malignancies, and other autoimmune conditions, AOSD remains a diagnosis of exclusion and poses considerable diagnostic challenges.^[3]^ The pathogenesis involves excessive production of pro-inflammatory cytokines, particularly interleukin (IL)-1, IL-6, IL-18, and tumor necrosis factor-alpha (TNF-α).^[4]^ These cytokines also underlie the joint involvement of AOSD, which is characterized by a distinct magnetic resonance imaging pattern and a synovial transcriptomic profile enriched for IL-1, IL-6, TNF, and ferritin-related genes.^[5]^ A hallmark of AOSD is hyperferritinemia, with serum ferritin levels often exceeding 10,000 ng/mL during active disease.^[6]^

Chronic systemic inflammation is a well-established risk factor for bone loss. Proinflammatory cytokines promote osteoclastogenesis through the receptor activator of nuclear factor kappa-B ligand (RANKL) pathway while inhibiting osteoblast function.^[7]^ This inflammation-mediated bone loss is well documented in rheumatoid arthritis and ankylosing spondylitis.^[8,9]^

Trabecular bone score (TBS), a textural analysis of lumbar spine dual-energy x-ray absorptiometry (DXA) images, provides information on bone microarchitecture beyond bone mineral density (BMD) and has shown utility in conditions such as diabetes and glucocorticoid-induced osteoporosis (GIO).^[10,11]^ Given the high inflammatory burden and frequent glucocorticoid use in patients with AOSD, TBS may provide additional insights into bone health in this population.

Glucocorticoid-induced osteoporosis is a major concern in AOSD management because most patients require prolonged glucocorticoid therapy.^[12]^ However, the independent contribution of disease-related inflammation to bone loss in AOSD remains unexplored.

Despite the chronic inflammatory nature of AOSD and the widespread use of glucocorticoid therapy in its management, systematic data on bone health in AOSD are lacking. A literature search revealed only isolated case reports of vertebral osteoporosis in individual patients^[13]^ and a small case series of 2 patients receiving denosumab for GIO.^[14]^ To date, no study has examined the prevalence of osteoporosis in AOSD cohorts or investigated the relationship between AOSD-specific inflammatory markers (C-reactive protein [CRP] and ferritin) and bone parameters.

This study evaluated bone health parameters in patients with AOSD and investigated the relationships among disease-specific inflammatory markers, BMD, and bone microarchitecture.

## 2. Materials and methods

### 2.1. Ethics statement

This study was approved by the Institutional Review Board of Ajou University Hospital (approval number: AJOUIRB-DB-2025-611; approval date: December 4, 2025). The requirement for written informed consent was waived by the Institutional Review Board owing to the retrospective nature of the study and the use of de-identified data. All methods were performed in accordance with the principles of the Declaration of Helsinki (October 2024 revision).^[15]^

### 2.2. Study design and population

This retrospective cross-sectional study was conducted and reported in accordance with the Strengthening the Reporting of Observational Studies in Epidemiology (STROBE) guidelines for cross-sectional studies.^[16]^

We included consecutive patients diagnosed with AOSD according to the Yamaguchi criteria^[1]^ who underwent DXA at Ajou University Hospital between January 2010 and December 2023. DXA was performed as part of routine clinical care based on the treating physician’s judgment. Patients were excluded if they had conditions known to affect bone metabolism other than AOSD or glucocorticoid use, including primary hyperparathyroidism, hyperthyroidism, chronic kidney disease (estimated glomerular filtration rate [eGFR] < 30 mL/min/1.73 m^2^), malignancy, or concurrent use of bone-active medications other than calcium and vitamin D supplementation. Menopausal status was recorded for female patients.

Because AOSD is a rare disease and this was a retrospective analysis, no a priori sample size calculation was performed; instead, all consecutive eligible patients during the study period were included in order to maximize statistical power. A post hoc analysis indicated that the achieved sample size provided approximately 90% power to detect the observed correlation between CRP and TBS (*r* = −0.45) and approximately 65% power for the corresponding partial correlation adjusted for age, sex, and BMI (*r* = −0.34), at a 2-sided α of 0.05.

### 2.3. Clinical and laboratory assessment

Demographic and clinical data, including age, sex, body mass index (BMI), disease duration, disease subtype (monophasic, polycyclic, or chronic articular), and cumulative glucocorticoid dose, were obtained from electronic medical records. The AOSD clinical subtype was classified based on the disease course pattern. Laboratory parameters obtained at the time of DXA included CRP (mg/dL) and serum ferritin (ng/mL). Serum CRP was measured by an immunoturbidimetric assay, and serum ferritin by electrochemiluminescence immunoassay (ECLIA) on a Roche Cobas analyzer (Roche Diagnostics, Mannheim, Germany), in accordance with the manufacturer’s protocols, in the accredited central laboratory of Ajou University Hospital, which participates in external quality-assessment programs. The cumulative glucocorticoid dose was calculated as the total prednisolone-equivalent dose from the AOSD diagnosis to the DXA examination. Data on serum vitamin D concentrations and on vitamin D or calcium supplementation were not systematically recorded and therefore could not be analyzed as covariates.

Treatment history at the time of DXA was reviewed, including current use of glucocorticoids, conventional disease-modifying antirheumatic drugs (cDMARDs; methotrexate, hydroxychloroquine), and biologic agents (tocilizumab, TNF-α inhibitors). Prior use of anti-osteoporosis medications and vertebral fracture history were also recorded.

### 2.4. Bone densitometry

BMD was measured in the lumbar spine (L1–L4 or available vertebrae based on individual anatomy and exclusion criteria), femoral neck, and total hip using DXA (Lunar Prodigy; GE Healthcare, Chicago, IL). BMD values are reported in g/cm^2^. Vertebrae with focal abnormalities (fractures, severe degenerative changes, or surgical hardware) were excluded for lumbar spine measurements, and the average BMD of the evaluable vertebrae was used. Daily quality control was performed using a manufacturer-supplied phantom, and the densitometer was calibrated according to the manufacturer’s recommendations. The precision errors (%CV = standard deviation/mean × 100) for the lumbar spine BMD and femoral neck BMD were 0.87% and 0.93%, respectively. The least significant change (LSC) was 0.024 g/cm^2^ for the lumbar spine BMD and 0.026 g/cm^2^ for the femoral neck BMD.

Osteoporosis was defined as a *T*-score ≤−2.5 at any measured site, and osteopenia as a *T*-score between − 1.0 and − 2.5, according to World Health Organization criteria.^[17]^ For patients aged < 50 years and premenopausal women, *Z*-scores were used as recommended by the International Society for Clinical Densitometry (ISCD); a *Z*-score ≤−2.0 was classified as “low bone density for age.”^[18]^ In the present cohort, 14 patients were aged < 50 years. A sensitivity analysis restricted to patients aged ≥ 50 years was performed to address potential misclassification.

TBS was evaluated retrospectively using TBS iNsight version 3.0.2.0 (Med-Imaps, Plan-les-Ouates, Switzerland). The software used the raw spine DXA images for the same regions of measurement as those used to estimate the lumbar spine BMD, and the lumbar spine TBS was computed as the mean value of individual measurements for L1–L4. For TBS, the precision error and LSC were 1.408% and 0.039, respectively. TBS values > 1.350 were considered normal, 1.200 to 1.350 as partially degraded, and < 1.200 as degraded.^[19]^

### 2.5. Statistical analysis

Continuous variables were expressed as mean ± standard deviation (SD) or median (interquartile range [IQR]), depending on distribution assessed by the Shapiro–Wilk test. Categorical variables were expressed as numbers and percentages. Spearman correlation coefficients with 95% confidence intervals (CIs), calculated using the Fisher *z*-transformation, were used to assess the relationship between inflammatory markers and bone parameters.

Given the number of correlation tests performed (20 tests across 5 predictor and 4 outcome variables), Bonferroni correction was applied to control for multiple comparisons, with corrected *P* < .05 considered significant.

Partial Spearman correlations adjusting for age were calculated to assess whether associations between inflammatory markers and bone parameters were independent of age. Additional partial correlations adjusting for age, sex, and BMI were performed for key relationships.

Ferritin and cumulative glucocorticoid doses were log10-transformed because of their markedly right-skewed distributions. CRP (mg/dL) was analyzed on the original scale, as the nonparametric Spearman correlation and odds ratio (OR) per 1-unit increase were considered interpretable without transformation.

Logistic regression analysis was performed to identify factors associated with osteoporosis. The multivariable models were adjusted for age, sex, and BMI. Each inflammatory marker was entered separately into the multivariable model to minimize variable load relative to the number of events. With 22 osteoporosis cases, the commonly used guideline of approximately 10 events per variable was met for models containing 1 inflammatory marker and 3 adjustment covariates.^[20]^ Subgroup analyses were performed for patients aged ≥ 50 years and for females aged ≥ 50 years; given the reduced sample sizes, these results should be considered hypothesis-generating. Statistical significance was set at *P* < .05. All analyses were performed using Python (version 3.11; Python Software Foundation) with the SciPy and StatsModels packages.

## 3. Results

### 3.1. Patient characteristics

**Figure 1.**
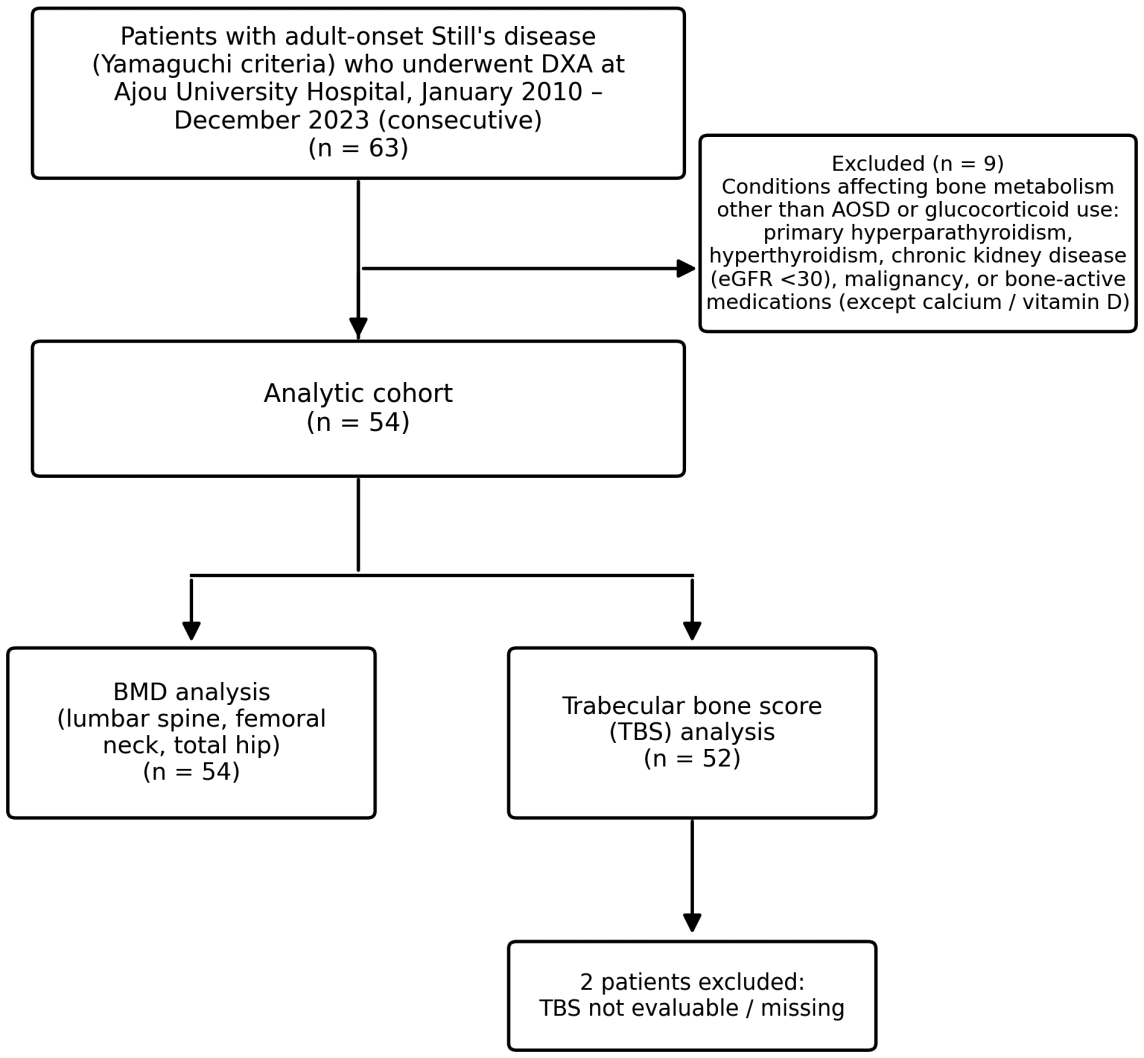
Flowchart of participant selection. AOSD = adult-onset Still’s disease, BMD = bone mineral density, CKD = chronic kidney disease, DXA = dual-energy x-ray absorptiometry, eGFR = estimated glomerular filtration rate, TBS = trabecular bone score.

The process of participant selection is summarized in Figure 1. Of 63 patients with AOSD identified in the hospital database who underwent DXA, 9 were excluded on the basis of the predefined criteria, yielding a final analytic cohort of 54 patients (Table 1). The mean age was 58.2 ± 12.0 years, and 43 patients (79.6%) were female. Among female patients, 29 (67.4%) were aged ≥ 50 years (used as a proxy for postmenopausal status). Mean BMI was 22.9 ± 2.8 kg/m^2^. Median disease duration from AOSD diagnosis to DXA examination was 29.5 months (IQR, 4.5–83.5). At the time of DXA, the median CRP was 0.14 mg/dL (IQR, 0.04–1.51), and the ferritin level was 128.8 ng/mL (IQR, 49.2–373.3). The median cumulative glucocorticoid dose was 5062.5 mg of prednisolone equivalents (IQR, 1181.2–9690.0).

**Table 1:** Baseline characteristics of the study population (N = 54).

Variable	Value
Demographics	
Age, yr	58.2 ± 12.0
Female, n (%)	43 (79.6)
Females aged ≥ 50 yr, n/total female (%)	29/43 (67.4)
BMI, kg/m^2^	22.9 ± 2.8
Disease duration, mo, median (IQR)	29.5 (4.5–83.5)
AOSD subtype, n (%)	
Monophasic	12 (22.2)
Polycyclic	7 (13.0)
Chronic articular	26 (48.1)
Unclassified	9 (16.7)
Laboratory findings at DXA, median (IQR)	
CRP, mg/dL	0.14 (0.04–1.51)
Ferritin, ng/mL	128.8 (49.2–373.3)
Cumulative glucocorticoid dose, mg	5062.5 (1181.2–9690.0)
Treatment at DXA, n (%)	
Current glucocorticoid use	42 (77.8)
cDMARDs	41 (75.9)
Methotrexate	25 (46.3)
Hydroxychloroquine	11 (20.4)
Biologic agents	8 (14.8)
Tocilizumab	7 (13.0)
TNF-α inhibitors	1 (1.9)
Prior anti-osteoporosis therapy	7 (13.0)
Prevalent vertebral fracture	3 (5.6)
Bone densitometry, mean ± SD	
Lumbar spine BMD, g/cm^2^	0.970 ± 0.206
Femoral neck BMD, g/cm^2^	0.769 ± 0.139
Total hip BMD, g/cm^2^	0.819 ± 0.147
TBS (n = 52)	1.404 ± 0.081
Bone status, n (%)	
Osteoporosis	22 (40.7)
Osteopenia	21 (38.9)
Normal	11 (20.4)
TBS category (n = 52), n (%)	
Normal (>1.350)	38 (73.1)
Partially degraded (1.200–1.350)	14 (26.9)
Degraded (<1.200)	0 (0.0)

Data are presented as mean ± SD, median (IQR), or n (%).

AOSD = adult-onset Still’s disease, BMD = bone mineral density, BMI = body mass index, cDMARDs = conventional disease-modifying antirheumatic drugs, CRP = C-reactive protein, DXA = dual-energy x-ray absorptiometry, IQR = interquartile range, SD = standard deviation, TBS = trabecular bone score, TNF-α = tumor necrosis factor-alpha.

Regarding AOSD clinical subtypes, 12 patients (22.2%) had monophasic disease, 7 (13.0%) had polycyclic disease, and 26 (48.1%) had chronic articular disease; 9 patients (16.7%) could not be classified. At the time of DXA, 42 patients (77.8%) were receiving glucocorticoids, 41 (75.9%) were on cDMARDs (methotrexate 25 [46.3%], hydroxychloroquine 11 [20.4%]), and 8 (14.8%) were receiving biologic agents (tocilizumab 7 [13.0%], TNF-α inhibitors 1 [1.9%]). Seven patients (13.0%) had received prior anti-osteoporosis therapy, and 3 (5.6%) had prevalent vertebral fractures.

### 3.2. Bone health parameters

Mean BMD values were 0.970 ± 0.206 g/cm^2^ at the lumbar spine, 0.769 ± 0.139 g/cm^2^ at the femoral neck, and 0.819 ± 0.147 g/cm^2^ at the total hip. Mean TBS was 1.404 ± 0.081. Based on T-score criteria, 22 patients (40.7%) had osteoporosis, 21 (38.9%) had osteopenia, and 11 (20.4%) had normal bone density, yielding an overall abnormal BMD rate of 79.6%. Among the 52 patients with available TBS data, 38 (73.1%) had normal microarchitecture (TBS > 1.350) and 14 (26.9%) had partially degraded microarchitecture (TBS 1.200–1.350); no patients had degraded microarchitecture (TBS < 1.200).

### 3.3. Correlations between inflammatory markers and bone parameters

**Figure 2.**
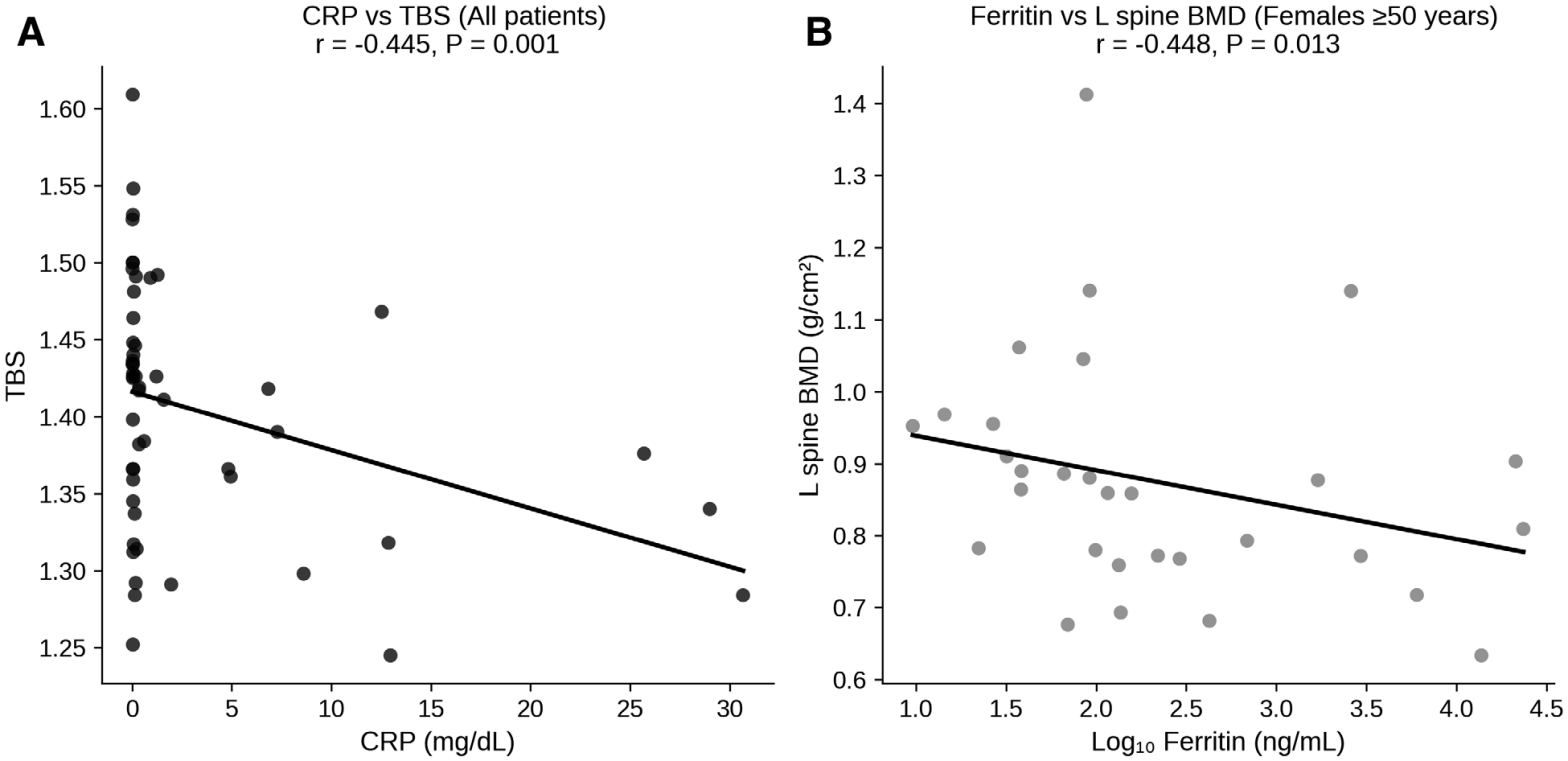
Scatter plots showing correlations between inflammatory markers and bone parameters. (A) CRP versus TBS in all patients (*r* = −0.445, *P* = .001; age-adjusted partial *r* = −0.352, *P* = .010). (B) Log-transformed ferritin versus lumbar spine BMD in women aged ≥ 50 years (*r* = −0.516, *P* = .004). BMD = bone mineral density, CRP = C-reactive protein, TBS = trabecular bone score.

Spearman correlation analysis revealed significant associations between inflammatory markers and bone parameters (Table 2). CRP showed a significant negative correlation with TBS (*r* = −0.445, 95% CI − 0.640 to − 0.196, *P* = .001; Fig. 2A), which remained significant after Bonferroni correction for 20 comparisons (adjusted *P* = .019). Age was negatively correlated with TBS (*r* = −0.533, 95% CI − 0.703 to − 0.304, *P* < .001; Bonferroni-adjusted *P* = .001). CRP also showed a negative trend with lumbar spine BMD (*r* = −0.264, *P* = .054). Log-transformed ferritin showed a trend toward a negative correlation with TBS (*r* = −0.257, *P* = .066, Table 3). The cumulative glucocorticoid dose was not significantly correlated with any bone parameters.

**Table 2:** Spearman correlations between inflammatory markers and bone parameters.

Variable	Lumbar spine BMD	Femoral neck BMD	Total hip BMD	TBS
Age	−0.142 (.305)	−0.307 (.024)*	−0.171 (.215)	−0.533 (<.001)†
CRP	−0.264 (.054)	−0.042 (.765)	−0.023 (.870)	−0.445 (.001)†
Log ferritin	−0.207 (.133)	−0.069 (.620)	0.091 (.512)	−0.257 (.066)
Log cumul. steroid	0.102 (.461)	−0.030 (.829)	−0.144 (.300)	0.230 (.101)
Disease duration	0.126 (.362)	−0.095 (.496)	−0.204 (.138)	0.263 (.060)

Data are Spearman *r (P* value).

BMD = bone mineral density, CRP = C-reactive protein, TBS = trabecular bone score.

† Significant after Bonferroni correction for 20 comparisons (adjusted *P* < .05).

* *P* < .05.

**Table 3:** Partial correlations between inflammatory markers and TBS.

Variable	Unadjusted *r (P*)	Age-adjusted *r (P*)	Age + Sex + BMI-adjusted *r (P*)
CRP	−0.445 (.001)*	−0.352 (.010)*	−0.336 (.015)*
Log ferritin	−0.257 (.066)	−0.173 (.219)	−0.159 (.260)
Log cumul. steroid	0.230 (.101)	−0.039 (.783)	−0.054 (.703)

Partial Spearman correlations.

BMI = body mass index, CRP = C-reactive protein, TBS = trabecular bone score.

* *P* < .05.

To evaluate whether the CRP–TBS association was independent of age, partial correlation analysis was performed. After adjusting for age, the CRP–TBS correlation remained significant (*r* = −0.352, *P* = .010). Furthermore, partial correlation adjusting for age, sex, and BMI confirmed that CRP was independently associated with TBS (*r* = −0.336, *P* = .015). In contrast, the log-transformed ferritin–TBS correlation was attenuated and no longer significant after adjusting for age (*r* = −0.173, *P* = .219) or age, sex, and BMI (*r* = −0.159, *P* = .260).

### 3.4. Logistic regression analysis for osteoporosis

In univariable logistic regression analysis (Table 4), CRP was significantly associated with osteoporosis (OR, 1.13; 95% CI, 1.01–1.27; *P* = .035). Log-transformed ferritin levels showed a trend toward an association (OR, 1.82; 95% CI, 0.94–3.55; *P* = .077). Age, BMI, disease duration, and the cumulative steroid dose were not significantly associated with osteoporosis.

**Table 4:** Logistic regression analysis for osteoporosis (N = 54).

Variable	OR (95% CI)	*P* value
Univariable		
Age	1.00 (0.96–1.05)	.916
Female	2.11 (0.49–9.06)	.315
BMI	0.93 (0.76–1.14)	.466
CRP	1.13 (1.01–1.27)	.035*
Log ferritin	1.82 (0.94–3.55)	.077
Log cumulative steroid	0.76 (0.54–1.07)	.119
Disease duration	1.00 (0.99–1.01)	.984
ESR at BMD	1.02 (1.00–1.04)	.083
Multivariable†		
CRP	1.13 (1.01–1.27)	.039*
Log ferritin	1.92 (0.94–3.91)	.073
Log cumulative steroid	0.75 (0.51–1.11)	.152
ESR at BMD	1.02 (0.99–1.04)	.129

BMD = bone mineral density, BMI = body mass index, CI = confidence interval, CRP = C-reactive protein, ESR = erythrocyte sedimentation rate, OR = odds ratio.

† Each inflammatory marker was entered separately into the multivariable model with age, sex, and BMI as covariates.

* *P* < .05.

In multivariable analysis (Table 4) adjusted for age, sex, and BMI, CRP remained independently associated with osteoporosis (OR, 1.13; 95% CI, 1.01–1.27; *P* = .039). Log-transformed ferritin showed a trend (OR, 1.92; 95% CI, 0.94–3.91; *P* = .073). The cumulative glucocorticoid dose was not significantly associated with osteoporosis in either univariable or multivariable analyses.

### 3.5. Subgroup analyses

**Figure 3.**
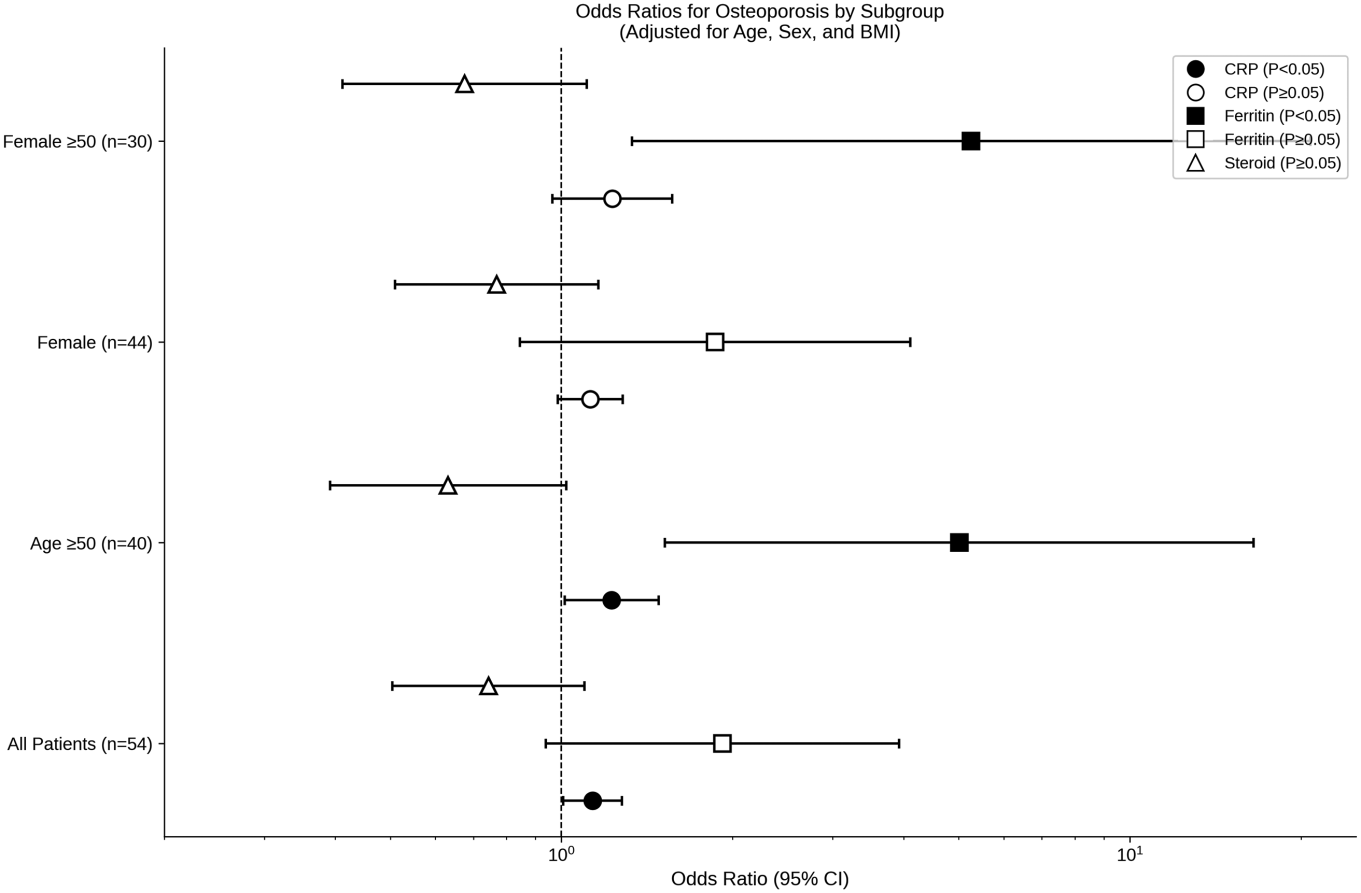
Forest plot showing odds ratios for osteoporosis by inflammatory markers across subgroups. Models adjusted for age and BMI (sex excluded in female-only subgroups). Filled markers indicate *P* < .05. BMI = body mass index, CI = confidence interval, CRP = C-reactive protein, OR = odds ratio.

In the subgroup of patients aged ≥ 50 years (n = 40; Table 5, Fig. 3), log-transformed ferritin was independently associated with osteoporosis after adjustment for age and BMI (OR, 4.34; 95% CI, 1.43–13.13; *P* = .009), and CRP remained significantly associated (OR, 1.22; 95% CI, 1.01–1.48; *P* = .037). In females aged ≥ 50 years (n = 29), log-transformed ferritin showed the strongest association with osteoporosis (OR, 5.73; 95% CI, 1.35–24.30; *P* = .018). In this subgroup, ferritin was also significantly correlated with lumbar spine BMD (*r* = −0.516, *P* = .004; Fig. 2B) and TBS (*r* = −0.419, *P* = .024). However, the wide CIs in these subgroup analyses reflect the limited sample sizes, and these findings should be interpreted as hypothesis-generating.

**Table 5:** Subgroup analysis of patients aged ≥ 50 years.

Subgroup	n	Variable	OR (95% CI)	*P* value
Age ≥ 50 yr	40	CRP	1.22 (1.01–1.48)	.037*
		Log ferritin	4.34 (1.43–13.13)	.009*
		Log cumulative steroid	0.63 (0.39–1.00)	.051
Female ≥ 50 yr	29	CRP	1.22 (0.96–1.55)	.103
		Log ferritin	5.73 (1.35–24.30)	.018*
		Log cumulative steroid	0.69 (0.42–1.14)	.150

Models adjusted for age and BMI (sex excluded in female-only subgroups). Each inflammatory marker was entered separately.

BMI = body mass index, CI = confidence interval, CRP = C-reactive protein, OR = odds ratio.

* *P* < .05. These results should be interpreted as hypothesis-generating given the limited sample sizes.

In the age-stratified analysis, osteoporosis prevalence was 21.4% (3/14) in patients aged < 50 years compared with 47.5% (19/40) in those aged ≥ 50 years (*P* = .119 by Fisher exact test). Among AOSD clinical subtypes, the chronic articular type showed the highest osteoporosis prevalence (53.8%, 14/26), followed by the monophasic type (33.3%, 4/12) and the polycyclic type (28.6%, 2/7), although the differences did not reach statistical significance (*P* = .280).

## 4. Discussion

To our knowledge, this study provides the first systematic real-world data on bone health in patients with AOSD. Our findings revealed a high prevalence of abnormal bone density and demonstrated that CRP was independently associated with impaired bone microarchitecture, as reflected by TBS, even after adjustment for age, sex, and BMI.

The high prevalence of osteoporosis and osteopenia in our AOSD cohort is notable and comparable to that observed in other chronic inflammatory rheumatic diseases such as rheumatoid arthritis.^[8]^ However, this prevalence may be overestimated, as DXA was performed based on clinical indications rather than as universal screening, potentially introducing selection bias toward patients with a higher a priori risk for osteoporosis.

The negative association between CRP and TBS was a key finding. Importantly, this association persisted in partial correlation analyses after adjustment for age and for age, sex, and BMI, indicating that the CRP–TBS relationship was not merely a reflection of age-related bone deterioration. TBS reflects bone microarchitecture and may be more sensitive than BMD alone to inflammation-induced deterioration in bone quality. CRP is a downstream marker of IL-6 activity, and IL-6 is a potent stimulator of osteoclastogenesis.^[7]^ The independent association between CRP and osteoporosis in multivariable analysis further supports the notion that systemic inflammation contributes to bone loss in AOSD, consistent with observations in other chronic inflammatory states. Nonetheless, a causal relationship cannot be established from this cross-sectional study.

The association between ferritin and osteoporosis, particularly in patients aged ≥ 50 years, is biologically plausible. Ferritin is not merely a passive marker of inflammation but also reflects iron storage. Iron overload disrupts bone homeostasis by inhibiting osteoblast differentiation while promoting osteoclastogenesis and bone resorption.^[21,22]^ In the subgroup of women aged ≥ 50 years, ferritin was correlated with both lumbar spine BMD and TBS, suggesting that its skeletal effect may become clinically relevant in the context of postmenopausal bone loss. In AOSD, the combination of hyperferritinemia and cytokine excess may create a synergistic environment that compromises bone integrity, although this hypothesis requires further investigation in prospective studies.

The cumulative glucocorticoid dose was not significantly associated with bone parameters. However, this should not be interpreted as evidence that glucocorticoids do not affect bone in patients with AOSD. The absence of a statistical association may reflect several methodological factors, including the limited sample size, the use of cumulative rather than time-weighted dose as the exposure metric, heterogeneous treatment patterns, and potential confounding by indication.^[23]^ The role of glucocorticoids in bone health in AOSD remains an open question requiring larger, prospective studies with more granular dosing data.

The observation that the chronic articular subtype of AOSD showed the highest osteoporosis prevalence is consistent with the concept that sustained inflammatory activity over time, rather than acute flares alone, may be the primary driver of bone loss. However, these differences were not statistically significant, likely reflecting limited statistical power.

This study adds to the limited body of literature on bone complications in AOSD by providing the first cohort-level data on BMD and bone microarchitecture in this population. Previous publications have highlighted the skeletal impact of systemic inflammatory diseases^[24,25]^; our findings extend these observations to AOSD, a disease characterized by uniquely high levels of ferritin and pro-inflammatory cytokines.

### 4.1. Clinical implications

These findings have several clinical implications. Bone health should be actively monitored in patients with AOSD, particularly those with persistently elevated inflammatory markers and those aged ≥ 50 years. Because TBS may capture inflammation-related deterioration in bone quality before it becomes apparent on BMD, incorporating TBS into routine DXA assessment could refine fracture-risk stratification in this population. Our data also support prioritizing effective control of systemic inflammation – rather than glucocorticoid minimization alone – as a component of skeletal protection, and they provide a rationale for considering earlier initiation of bone-protective therapy in high-risk patients. Whether tighter inflammatory control translates into a measurable bone benefit warrants confirmation in prospective studies.

### 4.2. Strengths and limitations

This study has several strengths. To our knowledge, it is the first to provide cohort-level data on both BMD and bone microarchitecture in AOSD, a rare disease for which only isolated case reports were previously available. The concurrent assessment of TBS allowed for the evaluation of bone quality that would be missed by densitometry alone. The robustness of the CRP–TBS association was reinforced by correction for multiple comparisons and by sequential adjustment for major confounders, and standardized DXA quality-control and precision procedures enhanced the reliability and reproducibility of the bone measurements.

This study also has several limitations. First, the cross-sectional design precludes establishing causality. Second, the study population was limited to AOSD patients who underwent DXA based on clinical indications rather than a standardized screening protocol, which may introduce selection bias. Third, CRP and ferritin were measured at a single time point and may not represent cumulative inflammatory burden; time-averaged or serial measurements would better capture long-term inflammatory exposure. Fourth, the multivariable models were adjusted only for age, sex, and BMI; several established osteoporosis risk factors (vitamin D levels, smoking, alcohol use, physical activity, and fracture history) were not available and therefore could not be included, potentially leading to residual confounding. Fifth, although 14 patients were aged < 50 years, our primary analysis applied T-score criteria uniformly; a sensitivity analysis restricted to patients aged ≥ 50 years supported the main findings. Sixth, in the subgroup analyses, the reduced sample sizes raise concerns about model stability and potential overfitting, as reflected by the wide CIs. Seventh, we lacked data on fracture outcomes beyond prevalent vertebral fractures. Finally, TBS data were unavailable for 2 patients.

## 5. Conclusion

These real-world data demonstrate a high prevalence of osteoporosis and osteopenia in patients with AOSD. CRP was independently associated with impaired bone microarchitecture (TBS) after adjustment for age, sex, and BMI, and with osteoporosis in multivariable logistic regression. Ferritin was significantly associated with osteoporosis in the subgroup of patients aged ≥ 50 years. These findings support routine bone health screening in AOSD, particularly in patients with elevated inflammatory markers and those aged ≥ 50 years. Larger, prospective studies incorporating serial inflammatory marker measurements, comprehensive confounder adjustment, and fracture outcome data are needed to confirm these associations.

## Author contributions

**Conceptualization:** Yong Jun Choi, Hyoun-Ah Kim.

**Data curation:** Yong Jun Choi, Ji-Won Kim, Ju-Yang Jung, Chang-Hee Suh.

**Formal analysis:** Yong Jun Choi.

**Investigation:** Ji-Won Kim, Ju-Yang Jung, Chang-Hee Suh.

**Methodology:** Yong Jun Choi.

**Resources:** Hyoun-Ah Kim.

**Supervision:** Hyoun-Ah Kim.

**Writing – original draft:** Yong Jun Choi.

**Writing – review & editing:** Ji-Won Kim, Ju-Yang Jung, Chang-Hee Suh, Hyoun-Ah Kim.
